# Analysis of Cardiovascular and Cerebrovascular Prognosis and Risk Factors in Patients with Primary Membranous Nephropathy

**DOI:** 10.34067/KID.0000000739

**Published:** 2025-02-21

**Authors:** Nan Ye, Lei Yang, Guoqin Wang, Wenrong Cheng, Bing Xie, Hongrui Dong, Lingqiang Kong, Xiaoyi Zhao, Yanqiu Geng, Hong Cheng

**Affiliations:** 1 Renal Division, Beijing Anzhen Hospital, Capital Medical University, Beijing, China; 2 Renal Division, Affiliated Hospital of Chifeng University, Neimenggu, China; 3 Renal Division, Third Medical Center, Chinese PLA General Hospital, Beijing, China

**Keywords:** cardiovascular events, membranous nephropathy

## Abstract

**Key Points:**

- Higher serum phospholipase A2 receptor antibody is an independent risk factor of arterial and venous thromboembolic events in patients with primary membranous nephropathy.
- The inflammatory system may play a role in this relationship.

**Background:**

This study investigated risk factors and possible mechanisms of arterial and venous thromboembolic events in patients with primary membranous nephropathy (pMN).

**Methods:**

Patients with pMN confirmed by renal biopsy from June 1, 2010, to March 3, 2023, were included, and the study outcome was set as a composite end point of acute coronary syndrome, heart failure, cerebral infarction, arrhythmia, pulmonary embolism, deep venous thrombosis, and all-cause mortality.

**Results:**

A total of 433 pMN patients with complete data were included, with a median follow-up time of 73 (45.5–101.6) months and a composite end point rate of 10.2%. We divided all patients with events into an early event group (events occurring in the first 2 years after renal biopsy) and a late event group (events occurring after 2 years after renal biopsy) according to the time of event. It showed a lower serum albumin and higher baseline value of serum phospholipase A2 receptor (PLA2R) antibody titer and mean value of each follow-up in the early event group compared with the late event group. Cox proportional hazards model showed that after adjusting for confounding factors, in addition to older age, history of deep vein thrombosis and higher urinary protein, higher baseline serum PLA2R antibody titers (odds ratio [OR], 1.034; 95% confidence interval [CI], 1.006 to 1.063; *P* = 0.015), and high high-sensitivity C-reactive protein level (OR, 1.049; 95% CI, 1.002 to 1.098; *P* = 0.041), renal pathology with segmental sclerotic lesions (OR, 3.480; 95% CI, 1.338 to 9.050; *P* = 0.011) were also independent risk factors for the occurrence of end point events. The results also showed that baseline serum IL-6 levels were significantly higher in the event group compared with the nonevent group (4.25 versus 3.21 pg/ml), and the difference was statistically significant (*P* = 0.009).

**Conclusions:**

In the early event group, higher serum PLA2R antibody and renal pathology with segmental sclerosis lesions may affect the occurrence of cardiovascular, cerebrovascular, and venous thrombotic events. The inflammatory system may play a role in this relationship.

## Visual Abstract

**Figure:**
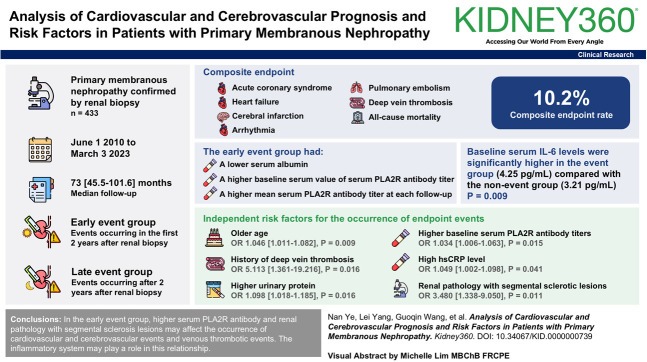

## Introduction

Primary membranous nephropathy (pMN) is one of the most common primary glomerular diseases in adults.^1^ We all focused on how to achieve remission of nephrotic syndrome (NS) as soon as possible and reduce the risk of progression to ESKD. However, the complications of pMN, especially the high incidence of thromboembolic complications, are also a problem that cannot be ignored and seriously threatens the survival of patients. pMN is the most likely to be associated with venous thromboembolic complications in all pathological types of NS,^2–4^ but the cause is not clear. In addition to venous thromboembolic complications, arterial thrombotic events resulting in cardiovascular events are also important complications. Patients with pMN are predominantly middle-aged and elderly and are at high risk of cardiovascular disease; occurrence of pMN contributes to their significantly increased risk of cardiovascular events. This study intends to investigate the epidemiological characteristics and risk factors of cardiovascular and cerebrovascular events and venous thrombotic events in patients with pMN.

## Methods

### Research Population

This was a retrospective cohort study. Patients with a definite diagnosis of pMN who underwent renal biopsy at Beijing Anzhen Hospital, Capital Medical University, between June 1, 2010, and March 1, 2023, were included in the study. Exclusion criteria were secondary membranous nephropathy, combined with other immune glomerulopathies, and incomplete follow-up data.

Institutional Review Board approval was granted for the aggregate dataset for research and quality improvement by the Ethics Committee of Beijing Anzhen Hospital, Capital Medical University. This study was conducted in accordance with the Declaration of Helsinki.

### Research Outcome

The study outcome was set as a composite of cardiovascular and cerebrovascular events and venous thrombotic events, specifically acute coronary syndrome, heart failure, arrhythmia, cerebral infarction, pulmonary embolism, deep vein thrombosis (DVT), and all-cause mortality. Acute coronary syndrome is defined as unstable angina or acute myocardial infarction.^5^ Heart failure includes heart failure with reduced ejection fraction, heart failure with mildly reduced ejection fraction, and heart failure with preserved ejection fraction.^6^ We divided all patients who had an end point event into an early event group (event occurred in the first 2 years after renal biopsy) and a late event group (event occurred after 2 years after renal biopsy) according to the time of event.

### Data Collection and Definitions

Baseline data were based on the most recent results before renal biopsy, and data on demographic characteristics, medical history, physical examination, and laboratory tests were collected. The pathological features of renal biopsy, immunohistochemical results of target antigen in membranous nephropathy, and treatment options were also collected. eGFR was calculated according to the CKD Epidemiology Collaboration formula. Serum M-type phospholipase A2 receptor (PLA2R) antibodies were detected by ELISA (EUROIMMUN, Germany) using serum specimens stored at −80°C. IL-6 was detected using serum samples stored at −80°C and detected using chemiluminescence (BECKMAN). Follow-up data were collected according to the frequency of approximately every 3 months, mainly 24-hour urinary protein, serum albumin, eGFR, and serum PLA2R antibodies.

### Statistical Analysis

Data were statistically analyzed using SPSS 27.0 software. Normally distributed measurement data were presented in the form of *x*±*s*, and comparisons between the two groups were performed using independent sample *t* test; non-normally distributed measurement data were presented in the form of *M* (*P* 25, *P* 75), and comparisons between the two groups were performed using Mann–Whitney *U* test; enumeration data were presented as the number of cases and percentage, and comparisons between the two groups were performed using chi-squared test or Fisher exact test. Risk factors affecting outcome were analyzed using Cox proportional hazards models. Subgroup analyses were used to further adjust for confounding factors in multivariate analyses, and interaction *P* values were calculated.

## Results

### Baseline Characteristics

A total of 433 individuals were included in the study, with a median follow-up time of 73 (45.5–101.6) months, a mean age of 52.1±14.0 years, and 254 men (58.7%). In terms of clinical characteristics, 277 patients (64.0%) had hypertension, 73 patients (16.9%) had diabetes mellitus, 27 patients (6.2%) had a history of coronary heart disease, two patients (0.5%) had a history of heart failure, 32 patients (7.4%) had a history of cerebrovascular disease, 13 patients (3.0%) had a history of DVT, 22 patients (5.1%) had a history of pulmonary embolism, and the mean baseline eGFR was 97.3±22.0 ml/min per 1.73 m^2^. Most of the patients had NS (77.1%), the median serum PLA2R antibody titer was 44.0 (4.9–117.6) relative unit (RU)/ml, median high-sensitivity C-reactive protein (hsCRP) was 0.67 (0.34–1.83) mg/L, median serum IL-6 was 3.31 (2.01–5.21) pg/ml, and median D-dimer was 215.5 (104.8–507.0) ng/ml. In terms of renal pathology, most of the patients were in membranous nephropathy stage 1 or 2 (88.7%). In terms of target antigen staining, all patients received PLA2R staining, of which 387 (89.4%) were PLA2R positive; 227 received thrombospondin type-1 domain-containing 7A staining, of which eight (3.5%) were thrombospondin type-1 domain-containing 7A positive; 124 received neural epidermal growth factor-like protein 1 staining, of which 14 (11.3%) were neural epidermal growth factor-like protein 1 positive; 77 received exostosin staining, of which 5 (6.5%) were exostosin positive. Immunofluorescence was dominated by Immunoglobulin G (IgG) deposition in all patients, of which 426 received IgG subclass staining, of which IgG4 deposition was predominant in 368 (86.4%), and 309 (71.4%) had C3 deposition. In terms of other pathological features, 83 (19.2%) had renal interstitial fibrosis, 30 (6.9%) had segmental sclerosis, 58 (13.4%) had ischemic lesions, and 58 (3.7%) had diabetic nephropathy, and 45 (10.4%) had obesity-associated glomerulomegaly.

### Incidence of End Point Events and Comparison of Baseline Characteristics

**Figure 1:**
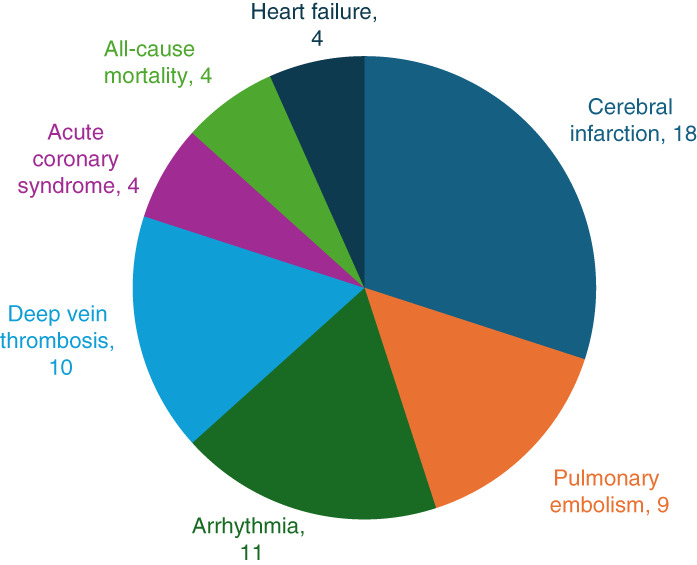
Number and distribution of events.

**Figure 2:**
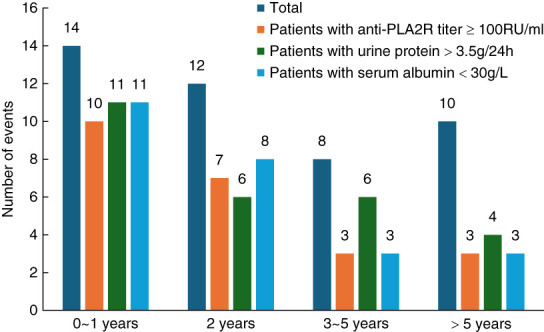
**Number of events over follow-up time.** The dark blue bar represents the total number of events. The orange bar represents the number of patients with serum PLA2R antibody titer ≥100 RU/ml at the time of event. The green bar represents the number of patients with urine protein >3.5 g/d at the time of event. The light blue bar represents the number of patients with serum albumin <30 g/L at the time of event. PLA2R, phospholipase A2 receptor; RU, relative unit.

The total composite end point number of events was 44, with an event rate of 10.2%, and the distribution of each event in the composite end point is shown in Figure 1. In comparison of baseline characteristics, the event group was older and had a higher proportion of men, more smokers, combined hypertension, more history of DVT, greater urinary protein, higher serum PLA2R antibody titer, higher hsCRP levels, and higher D-dimer and fibrin degradation product levels than the nonevent group (Table 1). There were 26 patients in the early event group and 18 patients in the late event group (Figure 2). Patients in the early event group had lower serum albumin, higher baseline serum PLA2R antibody titers, and higher mean values at each follow-up visit compared with patients in the late event group (Figure 2 and Table 2).

**Table 1:** Baseline characteristics

Variables	Total (*n*=433)	Event (*n*=44)	Nonevent (*n*=389)	*P* Value
Age, yr, mean (SD)	52.1 (14.0)	56.3 (11.6)	51.6 (14.1)	0.036
Male, No. (%)	254 (58.7)	33 (75.0)	221 (56.8)	0.020
Body mass index, kg/m^2^, mean (SD)	25.7 (3.7)	25.4 (3.2)	25.8 (3.8)	0.553
Systolic BP, mm Hg, mean (SD)	133.6 (18.1)	135.8 (12.8)	134.1 (19.4)	0.441
Diastolic BP, mm Hg, mean (SD)	82.2 (11.8)	82.1 (10.6)	82.4 (12.3)	0.888
Smoker, No. (%)	134/431 (31.1)	23/44 (52.3)	111/387 (28.7)	0.001
Hypertension, No. (%)	277 (64.0)	35 (79.5)	242 (62.2)	0.023
Diabetes, No. (%)	73 (16.9)	8 (18.2)	65 (16.7)	0.805
DVT history, No. (%)	13 (3.0)	4 (9.1)	9 (2.3)	0.013
Previous pulmonary embolism, No. (%)	22 (5.1)	2 (4.5)	20 (5.1)	0.865
Previous coronary artery disease, No. (%)	27 (6.2)	4 (9.1)	23 (5.9)	0.409
Heart failure history, No. (%)	2 (0.5)	1 (2.3)	1 (0.3)	0.486
Cerebrovascular disease history, No. (%)	32 (7.4)	5 (11.4)	27 (6.9)	0.288
Serum creatinine, *μ*mol/L, mean (SD)	71.7 (26.7)	77.1 (29.3)	71.1 (26.4)	0.155
eGFR, ml min^−1^ (1.73 m)^−2^, mean (SD)	97.3 (22.0)	91.6 (20.5)	97.9 (22.1)	0.070
24-h urine protein, g/d, mean (SD)	6.2 (4.1)	7.9 (5.0)	6.0 (4.0)	0.003
Albumin, g/L, mean (SD)	26.1 (7.2)	24.2 (6.6)	26.3 (7.2)	0.063
NS, No. (%)	334 (77.1)	40 (90.9)	294 (75.6)	0.022
Hemoglobin, g/L, mean (SD)	136.4 (18.8)	137.7 (18.7)	136.6 (18.7)	0.713
hsCRP, mg/L, median (IQR)	0.67 (0.34–1.83)	1.33 (0.52–5.11)	0.64 (0.33–1.63)	0.013
Serum IL-6, pg/ml, median (IQR)	3.31 (2.01–5.21)	4.25 (2.95–7.11)	3.21 (1.94–5.12)	0.009
Total cholesterol, mmol/L, mean (SD)	7.7 (2.6)	7.4 (2.0)	7.7 (2.7)	0.449
LDL-cholesterol, mmol/L, mean (SD)	4.9 (2.2)	5.0 (1.7)	4.9 (2.2)	0.677
Triglyceride, mmol/L, mean (SD)	2.5 (2.0)	2.3 (1.4)	2.5 (2.1)	0.418
C3, g/L, median (IQR)	1.19 (1.01–1.34)	1.25 (1.13–1.33)	1.19 (1.00–1.35)	0.116
C4, g/L, median (IQR)	0.27 (0.21–0.33)	0.30 (0.26–0.37)	0.26 (0.21–0.33)	0.013
D-dimer, ng/ml, median (IQR)	215.5 (104.8–507.0)	474.5 (216.8–985.8)	205.0 (96.0–449.0)	<0.001
FDP, *μ*g/ml, median (IQR)	2.05 (0.97–3.75)	3.70 (1.90–7.80)	1.90 (0.90–3.38)	<0.010
Serum PLA2R antibody titer, RU/ml, median (IQR)	44.0 (4.9, 117.6)	113.5 (9.9–312.9)	40.8 (4.3–103.5)	0.002
**Pathologic features, No. (%)**				
Pathological stage				0.852
*1*	162 (37.4)	17 (38.7)	145 (37.3)	
*2*	228 (52.7)	24 (54.5)	204 (52.4)	
*3*	43 (9.9)	3 (6.8)	40 (10.3)	
Immunofluorescence C3				0.573
*<2 +*	124 (28.6)	11 (25.0)	113 (29.0)	
*≥2 +*	309 (71.4)	33 (75.0)	276 (71.0)	
Renal interstitial fibrosis				0.181
*≤25%*	350 (80.8)	31 (70.5)	319 (82.0)	
*25% to ≤ 50%*	71 (16.4)	11 (25.0)	60 (15.4)	
*>50%*	12 (2.8)	2 (4.5)	10 (2.6)	
With segmental sclerotic lesions	30 (6.9)	6 (13.6)	24 (6.2)	0.065
With IgA mesangial area deposits	13 (3.0)	1 (2.3)	12 (3.1)	0.765
With ischemic renal impairment	48 (11.1)	5 (11.4)	43 (11.1)	0.951
Combined with diabetic nephropathy	16 (3.7)	3 (6.8)	13 (3.3)	0.247
Combined with obesity-related glomerulomegaly	45 (10.4)	3 (6.8)	42 (10.8)	0.412
PLA2R positive	387 (89.4)	41 (93.2)	346 (88.9)	0.387
THSD7A positive	8/227 (3.5)	1/19 (5.3)	7/208 (3.4)	0.668
NELL-1 positive	14/124 (11.3)	2/15 (13.3)	12/109 (11.0)	0.790
EXT	5/77 (6.5)	0/9 (0.0)	5/68 (7.4)	—

DVT, deep vein thrombosis; EXT, exostosin; FDP, fibrin degradation product; hsCRP, high-sensitivity C-reactive protein; IQR, interquartile range; NELL-1, neural epidermal growth factor-like protein 1; NS, nephrotic syndrome; PLA2R, phospholipase A2 receptor; RU, relative unit; THSD7A, thrombospondin type-1 domain-containing 7A.

**Table 2:** Comparison of the clinical characteristics at the time of event between the early event group and late event group

Variables	Total (*n*=44)	Early Event Group (*n*=26)	Late Events Group (*n*=18)	*P* Value
Age (yr)^a^	60.0 (52.3–70.3)	58.5 (52.0–65.8)	61.5 (52.5–73.0)	0.333
Serum creatinine (*μ*mol/L)^a^	87.4 (66.8–143.5)	86.4 (64.4–117.0)	100.5 (69.5–182.8)	0.358
eGFR (ml min^−1^ [1.73 m]^−2^)^a^	70.5±30.3	76.4±28.5	62.0±31.6	0.121
24-h urine protein (g/d)^a^	6355.5 (2073.7–9629.9)	6674.9 (2597.0–10177.7)	5291.6 (799.2–9394.8)	0.535
Albumin (g/L)^a^	28.9±9.4	25.9±8.9	33.3±8.4	0.008
Baseline serum PLA2R antibody titer (RU/ml)^b^	44.0 (4.9–117.6)	161.3 (72.4–371.9)	48.1 (0.0–253.1)	0.037
Serum PLA2R antibody titer (RU/ml)^a^	40.6 (5.7–206.7)	58.5 (14.4–237.3)	13.7 (0.0–114.0)	0.084
Mean value of each follow-up visit for serum PLA2R antibody titer (RU/ml)^c^	109.1 (23.4–249.8)	124.8 (68.7–358.5)	67.2 (6.3–157.7)	0.045

PLA2R, phospholipase A2 receptor; RU, relative unit.

a Characteristics at the time of event.

b Characteristics at baseline.

c Mean value of each follow-up visit for characteristics.

### Cox Regression Analysis

**Figure 3:**
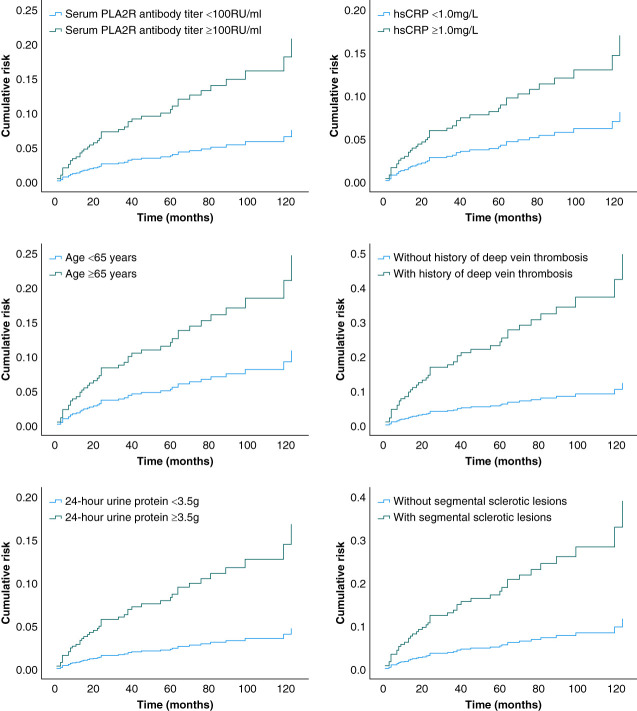
**Independent risk factors shown by a Cox proportional hazards model.** hsCRP, high-sensitivity C-reactive protein.

Univariate analysis showed that baseline serum PLA2R antibody titer was quite different between the event group and nonevent group; therefore, we performed Cox proportional hazards model analysis using baseline serum PLA2R antibody titer as the main analysis variable. The univariate Cox proportional hazards model (model 1), Cox proportional hazards model adjusted for clinical confounders (model 2), and Cox proportional hazards model adjusted for clinical and pathological confounders (model 3) all suggested that elevated baseline serum PLA2R antibody titer was an independent risk factor affecting the occurrence of end point events (Table 3). In addition, older age, history of DVT, higher urinary protein, higher hsCRP level, and presence of segmental sclerotic lesions were also independent risk factors for the occurrence of the end point event (Figure 3 and Table 3).

**Table 3:** Cox regression model

Variables	Model 1		Model 2		Model 3	
	HR (95% CI)	*P* Value	HR (95% CI)	*P* Value	HR (95% CI)	*P* Value
Serum PLA2R antibody titer (RU/ml, <50 for each 50 increase)	1.048 (1.023 to 1.073)	<0.001	1.036 (1.007 to 1.065)	0.013	1.034 (1.006 to 1.063)	0.015
Age, yr			1.037 (1.004 to 1.070)	0.027	1.046 (1.011 to 1.082)	0.009
Male			1.655 (0.714 to 3.836)	0.241	1.802 (0.765 to 4.246)	0.178
Smoker			1.707 (0.822 to 3.545)	0.151	1.617 (0.771 to 3.395)	0.204
Body mass index (kg/m^2^)			0.946 (0.862 to 1.039)	0.248	0.953 (0.861 to 1.055)	0.354
Hypertension			1.755 (0.780 to 3.944)	0.174	1.548 (0.668 to 3.584)	0.308
Diabetes			1.055 (0.449 to 2.476)	0.902	0.805 (0.288 to 2.252)	0.805
DVT history			3.533 (0.990 to 12.610)	0.052	5.113 (1.361 to 19.216)	0.016
Previous pulmonary embolism			0.491 (0.100 to 2.404)	0.380	0.340 (0.063 to 1.844)	0.211
Previous coronary artery disease			1.294 (0.409 to 4.099)	0.661	1.455 (0.429 to 5.922)	0.547
Heart failure history			1.116 (0.085 to 14.675)	0.934	0.967 (0.073 to 12.790)	0.980
Cerebrovascular disease history			0.836 (0.302 to 2.313)	0.730	0.762 (0.232 to 2.506)	0.654
eGFR (ml min^−1^ [1.73 m]^−2^)			1.010 (0.991 to 1.029)	0.312	1.018 (0.996 to 1.041)	0.101
24-h urine protein (g/d)			1.094 (1.015 to 1.180)	0.019	1.098 (1.018 to 1.185)	0.016
Albumin (g/L)			1.031 (0.966 to 1.100)	0.362	1.040 (0.970 to 1.114)	0.268
LDL-cholesterol (mmol/L)			0.929 (0.767 to 1.125)	0.451	0.941 (0.778 to 1.138)	0.529
hsCRP (mg/L)			1.053 (1.006 to 1.103)	0.026	1.049 (1.002 to 1.098)	0.041
**Pathologic features, No. (%)**						
Immunofluorescence C3						
*<2+*					1 (reference)	
*≥2+*					0.735 (0.336 to 1.609)	0.442
Renal interstitial fibrosis					1.597 (0.804 to 3.171)	0.181
With segmental sclerotic lesions					3.480 (1.338 to 9.050)	0.011
With IgA mesangial area deposits					1.104 (0.141 to 8.659)	0.925
With ischemic renal impairment					0.544 (0.115 to 2.583)	0.444
Combined hypertensive nephrosclerosis					3.651 (0.480 to 27.783)	0.211
Combined with diabetic nephropathy					2.727 (0.0.568 to 13.096)	0.210
Combined obesity-related glomerulomegaly					0.458 (0.124 to 1.698)	0.243

Model 1: univariate Cox proportional hazards model.

Model 2: adjusted for clinical factors.

Model 3: adjusted for clinical and pathological factors.

CI, confidence interval; DVT, deep vein thrombosis; HR, hazard ratio; hsCRP, high-sensitivity C-reactive protein; OR, odds ratio; PLA2R, phospholipase A2 receptor; RU, relative unit.

### Subgroup Analyses

Univariate analysis and Cox regression analysis adjusted for clinical and pathological confounders showed that elevated baseline serum PLA2R antibody titer was an independent risk factor for the occurrence of end point events, and to further confirm the reliability of this result, subgroup analysis and component interaction *P* values were performed. The results showed that, regardless of age, sex, smoking, presence of hypertension, diabetes, coronary heart disease, history of DVT, eGFR level, urinary protein level, hsCRP level, and renal pathology combined with segmental sclerosis lesions, the baseline value of serum PLA2R antibody titer had a consistent effect on the end point events, and its increase was an independent risk factor affecting the occurrence of the end point events (Table 4).

**Table 4:** Effect of baseline serum PLA2R antibody titer on end point events in different subgroups

Group	HR (95% CI)	Interaction *P* Value
**Age, yr**		0.386
Age <65	1.039 (1.005 to 1.075)	
Age ≥65	1.062 (1.024 to 1.101)	
**Sex**		0.141
Female	1.074 (1.032 to 1.117)	
Male	1.034 (1.002 to 1.067)	
**Smoker**		0.542
Yes	1.037 (1.005 to 1.070)	
No	1.053 (1.014 to 1.094)	
**History of hypertension**		0.898
Yes	1.044 (1.017 to 1.071)	
No	1.049 (0.979 to 1.123)	
**History of diabetes**		0.496
Yes	0.948 (0.707 to 1.271)	
No	1.050 (1.025 to 1.076)	
**History of coronary heart disease**		0.774
Yes	0.978 (0.603 to 1.588)	
No	1.050 (1.024 to 1.075)	
**History of deep venous thrombosis**		0.943
Yes	1.043 (0.942 to 1.154)	
No	1.047 (1.020 to 1.073)	
**eGFR, ml min** ^**−**^ **^1^ (1.73 m)** ^**−**^ **^2^**		0.595
≥60	1.049 (1.024 to 1.075)	
<60	0.999 (0.836 to 1.195)	
**24-h urine protein, g/d**		0.565
<3.5	0.355 (0.009 to 13.953)	
≥3.5	1.043 (1.017 to 1.069)	
**hsCRP, mg/L**		0.379
<1.0	1.057 (1.020 to 1.096)	
≥1.0	1.034 (1.000 to 1.069)	
**Segmental sclerotic lesions**		0.346
Yes	0.962 (0.798 to 1.159)	
No	1.053 (1.028 to 1.079)	

CI, confidence interval; HR, hazard ratio; hsCRP, high-sensitivity C-reactive protein; PLA2R, phospholipase A2 receptor.

## Discussion

The results of this study showed that the incidence of cardiovascular and cerebrovascular events and venous thrombotic events was 10.2% in patients with pMN; most of the end point events occurred in the first 2 years after diagnosis, and patients with events occurring in the first 2 years had lower serum albumin, higher baseline serum PLA2R antibody titers, and higher mean values at each follow-up compared with patients with events occurring after 2 years. The results of Cox regression analysis and subgroup analysis adjusted for confounding factors suggested that elevated baseline serum PLA2R antibody titer was an independent risk factor of cardiovascular, cerebrovascular, and venous thrombotic events in patients with pMN. In addition, hsCRP, an inflammatory marker, was also an independent risk factor affecting the occurrence of cardiovascular, cerebrovascular, and venous thrombotic events in patients with pMN, and another inflammatory marker IL-6 detected on this basis was also significantly increased in the event group, indicating that serum PLA2R antibody and inflammatory system may play a role in the occurrence of cardiovascular and cerebrovascular events and venous thrombotic events in patients with pMN.

Some previous studies have reported on cardiovascular outcomes in patients with pMN. Lee *et al.*^7^ conducted a retrospective cohort study to analyze the risk and risk factors of cardiovascular events in patients with pMN, and the results showed that independent risk factors for the occurrence of cardiovascular events in patients with pMN included advanced age, diabetes, history of cardiovascular events, and NS severity. Their findings showed that the incidence of cardiovascular events was at least as high as the incidence of ESKD in the first 2 years after pMN diagnosis and even exceeded the incidence of ESKD in patients with baseline eGFR ≥60 ml/min per 1.73 m^2^. Taking into account the risk of cardiovascular and cerebrovascular events, morbidity, long-term sequelae, and effect on quality of life, the authors concluded that prevention of cardiovascular events should be considered as an important early treatment goal in clinical practice. Another cohort study^8^ on Chinese patients analyzed the risk of arterial embolic events in 766 patients with pMN and found that the incidence of arterial embolic events was 9.3%, and advanced age was an independent risk factor affecting the occurrence of arterial embolic events. The results of a retrospective cohort study from New Zealand showed that the incidence of cardiovascular events in patients with pMN was 18.6%, and advanced age, male sex, diabetes, and no statin treatment were associated with the risk of cardiovascular events.^9^

Our study also analyzed the risk factors of cardiovascular and cerebrovascular events and venous thrombotic events in patients with pMN. In addition to advanced age, history of DVT, large urine protein similar to the results of previous studies, this study also found that high serum PLA2R antibody titer, elevated hsCRP, and renal pathology with segmental sclerosis lesions were also independent risk factors. Previous studies did not focus on membranous nephropathy target antigen, renal pathology, and inflammatory system problems. Previous studies on risk factor analysis for thromboembolic events in patients with NS or GN have shown that the more severe the NS (as shown by greater urinary protein and/or lower serum albumin levels), the higher the risk of thromboembolic events.^10,11^ Our results also demonstrate a relationship between high urinary protein and end point events. However, high serum PLA2R antibody titers and renal pathology with segmental sclerotic lesions tend to be associated with severe NS or treatment refractory of NS.^12,13^ Thus, the effect of high serum PLA2R antibody titers and renal pathology with segmental sclerotic lesions on end point events, particularly in patients whose events occurred in the first 2 years after renal puncture, may depend more on their effect on the severity of NS. However, it is important to note that patients in our study whose end point events occurred after 2 years had significantly reduced urinary protein and had significantly lower serum PLA2R antibody titers than baseline values, and the cause of the end point events in these patients may have been more influenced by the mechanism of thromboembolism itself rather than by the degree of NS. So, what are the mechanisms of this thromboembolism itself?

The results of this study showed that high hsCRP levels were also an independent risk factor affecting the occurrence of end point events, and further test results also showed that IL-6, an inflammatory indicator, was also significantly increased in the event group. Therefore, the inflammatory system may play a key role in this. hsCRP is a nonspecific marker of systemic inflammatory acute phase synthesized by the liver, where IL-6 is produced in large amounts, prompting the liver to synthesize large amounts of hsCRP. IL-6 is a cytokine with a wide range of biological effects and plays an important role in the innate immune system, which can be produced by macrophages, monocytes, endothelial cells, fibroblasts, podocytes, mesangial cells, and tubular epithelial cells. IL-6 receptors are mainly expressed on the surface of endothelial cells, in addition to podocytes.^14–16^ In addition to this, IL-6 has the following biological effects. First, IL-6 promotes atherosclerosis and plaque progression and destabilizes plaques.^15,17^ Second, IL-6 promotes coagulation, which induces an increase in peripheral blood platelet count, induces tissue factor expression on the surface of monocytes, initiates procoagulation by initiating the extrinsic coagulation pathway, induces plasminogen activator inhibitor type 1 production, inhibits the fibrinolytic system, increases fibrinogen production, and leads to thrombosis.^15,18^ Again, IL-6 also has the effect of inducing B-cell maturation and differentiation and secreting antibodies.^14,18–21^ Because IL-6 has such a wide range of biological effects and plays a key role in the development of atherosclerosis, therapeutic agents targeting it have emerged. The Trial to Evaluate Reduction in Inflammation in Patients With Advanced Chronic Renal Disease Utilizing Antibody Mediated IL-6 Inhibition study^22^ was a randomized, controlled, double-blind, phase 2 clinical trial in patients with moderate-to-severe CKD and hsCRP ≥2 mg/L to observe the efficacy and safety of ziltivekimab, a fully human mAb against IL-6 ligand. The study results showed that ziltivekimab significantly reduced levels of biomarkers of atherosclerosis-related inflammation and thrombosis, such as hsCRP, compared with placebo, which further demonstrated the important role of IL-6 in atherosclerosis and thrombosis. It is well known that PLA2R is the most common target antigen of pMN and is expressed on the surface of human podocytes.^23,24^ Anti-PLA2R antibodies bind to PLA2R to form subepithelial immune complexes, resulting in podocyte injury. So, does podocyte injury caused by anti-PLA2R antibody cause increased IL-6 production by podocytes, which in turn promotes atherosclerosis and thrombosis, thus increasing the risk of cardiovascular, cerebrovascular, and thromboembolic events in patients with pMN? Of course, this is only our hypothesis and needs to be further confirmed. However, this finding lays a foundation for our further study on the relationship between serum PLA2R antibodies and the inflammatory system and their role in cardiovascular and cerebrovascular prognosis.

This study has some limitations. First, the number of end point events was small, so arterial thromboembolic events and venous thromboembolic events were not analyzed separately, but as a composite end point, it is difficult to illustrate the difference between the two. Second, this study is a retrospective cohort study with many confounding factors that may be difficult to correct completely. Finally, the effect of many laboratory parameters on end point events may have changed over time. This study only considered changes over time in age, eGFR, urinary protein quantification, serum albumin, and serum PLA2R antibodies, but not other laboratory parameters, which may affect the analysis of the results.

In this study, we focused on cardiovascular, cerebrovascular, and venous thrombotic events in patients with pMN and analyzed the risk factors. It was found that in the first 2 years after renal puncture, high serum PLA2R antibody titer and renal pathology with segmental sclerosis lesions may affect the occurrence of cardiovascular, cerebrovascular, and venous thrombotic events through the severity of NS. While NS was relieved with prolonged follow-up time, anti-PLA2R antibody may affect the occurrence of cardiovascular and cerebrovascular events and venous thrombotic events by promoting the inflammatory response represented by IL-6. This lays a foundation for further study on the relationship between serum PLA2R antibody and inflammatory system and its role in cardiovascular and cerebrovascular prognosis.

## Data Availability

Partial restrictions to the data and/or materials apply. The datasets used and analyzed during the current study are available from the corresponding author on reasonable request.
